# Prevalence of atrial fibrillation and conduction abnormalities in Chagas disease: a systematic review and an updated meta-analysis

**DOI:** 10.1007/s10840-026-02243-8

**Published:** 2026-02-10

**Authors:** Francisco Rafael Gomes Lima, Kênnio Alves Aguiar, Aldo José Silva de Castro Costa, Anna Clara Lopes Bonfim Amorim, Giulia Valente Ribeiro, Guilherme Oliveira Gomes, Lays Beatrice Lima Matos Costa, Muhammad Sharjeel Abbas, Mrunalini Dandamudi, Juliana Giorgi, Warisha Kanwal, Carlos Eduardo Batista de Lima

**Affiliations:** 1 Department of Medicine, Federal University of Piauí, R. Dr. Anísio Maia, 1264, Bairro Ininga, Teresina, PI Brazil; 2 Gomal Medical College, Khyber Medical University, Peshawar, Khyber Pakhtunkhwa Pakistan; 3 Montefiore Einstein Medical Center, Bronx, NY USA; 4 Sirio Libanes Hospital, São Paulo, São Paulo Brazil; 5 Albert Einstein Hospital, São Paulo, São Paulo Brazil; 6 Karachi Medical And Dental College, Karachi, Pakistan

## Abstract

**Background:**

Chagas disease (CD) remains a significant public health problem and may progress to Chagas cardiomyopathy (ChCM), a distinct inflammatory–fibrotic myocardial phenotype characterized by a high burden of atrial fibrillation (AF) and conduction system abnormalities. Updated estimates of the prevalence of these electrical disturbances are essential to inform clinical risk stratification and guideline development.

**Objective:**

This updated meta-analysis aimed to evaluate the prevalence of AF and Conduction Abnormalities in ChCM.

**Methods:**

We conducted a comprehensive systematic review and meta-analysis of 33 studies published through February 2025, comprising 17,100 participants, of whom 6,644 had ChCM. The prevalence of AF and conduction abnormalities was evaluated in individuals with ChCM compared with non-Chagas controls, including healthy individuals and patients with CD and non-ChCM. Pooled estimates were generated using random-effects models, with formal assessment of between-study heterogeneity and publication bias. The review protocol was prospectively registered in PROSPERO (CRD420251005184).

**Results:**

AF was significantly more prevalent in ChCM (9.9%) than in controls (6.6%; OR = 1.88, 95% CI: 1.46–2.42). Among patients with cardiomyopathy, AF was more frequent in CD patients (12.1% vs. 9.5%; OR = 1.31, 95% CI: 1.07–1.60). Compared with ischemic cardiomyopathy, ChCM was associated with a higher AF risk (OR = 1.55; 95% CI: 1.13–2.12), whereas no difference was observed with dilated cardiomyopathy. Other conduction abnormalities were markedly more common in ChCM: first-degree atrioventricular block (OR = 2.71, 95% CI: 1.66–4.44), right bundle branch block (isolated or with left anterior fascicular block), and second-degree atrioventricular block.

**Conclusions:**

This updated meta-analysis demonstrates that Chagas cardiomyopathy is associated with a higher prevalence of atrial fibrillation and conduction abnormalities compared with other cardiomyopathy subtypes. These findings highlight the distinctive arrhythmogenic profile of Chagas cardiomyopathy and support the need for systematic electrocardiographic surveillance and heightened clinical awareness in affected patients. Future studies are warranted to define thromboembolic risk better, optimize rhythm management, and inform disease-specific strategies to reduce morbidity and mortality in populations affected by Chagas disease.

**Supplementary information:**

The online version contains supplementary material available at https://doi.org/10.1007/s10840-026-02243-8.

## Introduction

**Fig. 1:**
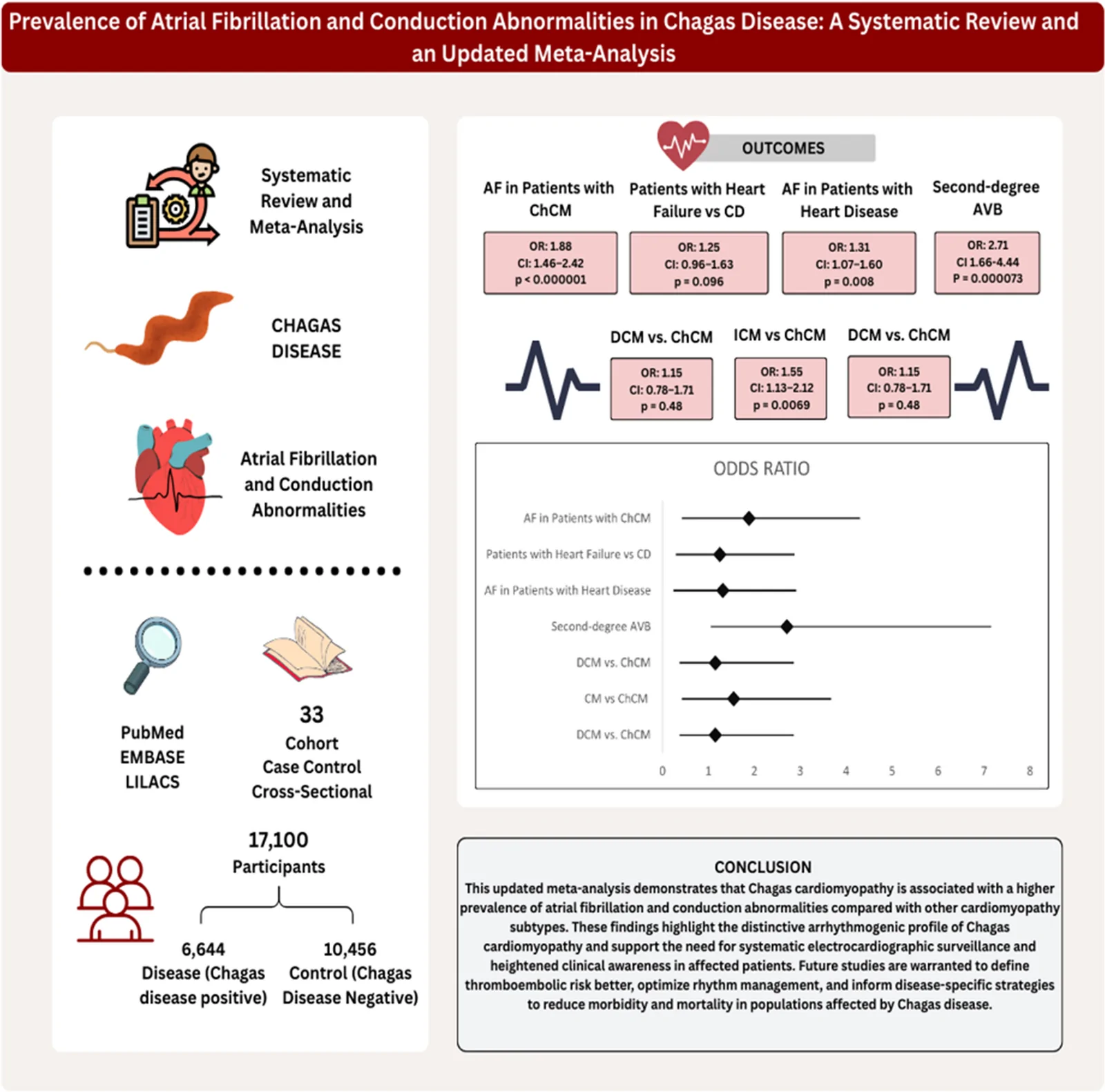
Graphical abstract of systematic review and meta-analysis evaluating the prevalence of atrial fibrillation and conduction abnormalities in chagas disease

Chagas disease (CD), a chronic parasitic infection caused by *Trypanosoma cruzi*, continues to represent a substantial public health burden in Latin America and has emerged as a growing global concern driven by international migration. Globally, approximately 7 million people are infected, with Brazil bearing the highest disease burden cases [1–3]. The natural history of CD is traditionally divided into an acute phase, followed by a prolonged indeterminate chronic phase, and, in a subset of patients, progression to a chronic determinate phase with cardiac and/or gastrointestinal involvement [4] (Fig. 1).

The most severe chronic manifestation of CD is Chagas cardiomyopathy (ChCM), a distinct arrhythmogenic myocardial phenotype characterized by conduction system involvement, atrial and ventricular arrhythmias, and progressive heart failure. These electrical abnormalities, particularly atrial fibrillation (AF) and atrioventricular or bundle branch block (BBB), are major contributors to thromboembolic events, sudden cardiac death, and overall morbidity [4, 5].

Previous meta-analyses, including that by Rojas et al. (2018), have established that individuals with ChCM exhibit a higher prevalence of conduction abnormalities than the general population, thereby confirming the arrhythmogenic nature of the disease [6, 7]. However, existing syntheses primarily relied on comparisons with healthy or seronegative controls. They did not systematically evaluate whether the arrhythmic burden observed in ChCM differs from that seen in other major cardiomyopathy subtypes. As a result, the relative contribution of ChCM to AF and conduction system disease within the broader spectrum of structural heart disease has remained insufficiently characterized. In the context of increasing global migration and the expanding geographic distribution of CD, an updated, methodologically refined meta-analysis was warranted. By integrating contemporary data and directly comparing ChCM with ischemic, dilated, and idiopathic cardiomyopathies, the present study provides a more nuanced and clinically relevant assessment of electrophysiological abnormalities in this challenging population, thereby addressing a critical gap in the existing literature.

## Materials and methods

### Search strategy

We systematically searched PubMed, EMBASE, and LILACS for studies that met the inclusion criteria and were published through February 2025 using the following search strategy: (“Chagas Disease” [MeSH] OR “Chagasic cardiomyopathy” OR “Chagas Disease” OR “Trypanosoma cruzi infection” OR “Chagas Cardiomyopathy” OR “Chagas Cardiomyopathy” [MeSH]) AND (“Atrial Fibrillation” [MeSH] OR “Atrial Fibrillation” OR “Arrhythmias, Cardiac” [MeSH] OR “Arrhythmias Cardiac” OR “heart failure” OR Electrocardiographic OR electrocardiogram OR “stroke risk factor”) AND (“ischemic cardiomyopathy” OR “non-chagasic” OR nonchagasic OR seronegative OR T. cruzi-noninfected OR “negative serology” OR non-chagas OR “primary DCM” OR “idiopathic disease” OR noninfected OR “other DCM”). Additionally, we searched clinical trial registries (e.g., ClinicalTrials.gov, WHO ICTRP) to identify unpublished or ongoing studies, in accordance with PRISMA recommendations. Study selection was performed independently by two authors (FL and LC), with disagreements resolved by a third author (KA).

### Eligibility criteria

We included studies reporting data on AF and/or conduction abnormalities, such as atrioventricular block (AVB), BBB, or intraventricular conduction delay, in patients with serologically confirmed Chagas disease, regardless of the serological technique used. Eligible studies were required to provide sufficient data to assess the prevalence or frequency of these electrophysiological abnormalities.

Studies were excluded if they were case reports or case series with fewer than 10 participants, abstract-only publications or conference proceedings without accessible full text, or narrative reviews and editorials lacking original patient-level data. Additionally, studies were excluded when they lacked a control group, did not report outcomes of interest, failed to confirm the diagnosis of Chagas disease, or involved overlapping study populations.

Throughout this manuscript, *CD* denotes individuals with positive serology for *Trypanosoma cruzi*, whereas *ChCM* denotes patients with CD and established structural heart disease or systolic dysfunction.

For subgroup analyses, cardiomyopathies were defined according to established clinical and etiologic criteria. The presence of left ventricular systolic dysfunction attributable to significant coronary artery disease or prior ischemic myocardial injury is described as ischemic cardiomyopathy (ICM). Dilated cardiomyopathy (DCM) was characterized by ventricular dilatation with impaired systolic function not explained by coronary artery disease or primary valvular pathology. Idiopathic dilated cardiomyopathy (IDCM) refers to a non-ischemic, non-Chagas, and non-valvular dilated cardiomyopathy of unknown etiology. The ChCM was defined by chronic structural myocardial involvement and conduction system abnormalities occurring in patients with serologically confirmed *Trypanosoma cruzi* infection.

Most included studies reported AF as a binary outcome based on electrocardiographic findings or medical record documentation, without consistent differentiation between paroxysmal, persistent, or permanent AF. Likewise, AF detection methods varied across studies and included resting electrocardiogram, Holter monitoring, hospital discharge diagnoses, or prior clinical history. Due to inconsistent reporting, stratified analyses by AF subtype or detection method were not feasible.

Data regarding anticoagulant therapy use among patients with AF were inconsistently reported across studies and could not be reliably synthesized or compared between Chagas and non-Chagas groups.

### Study selection and characteristics

**Fig. 2:**
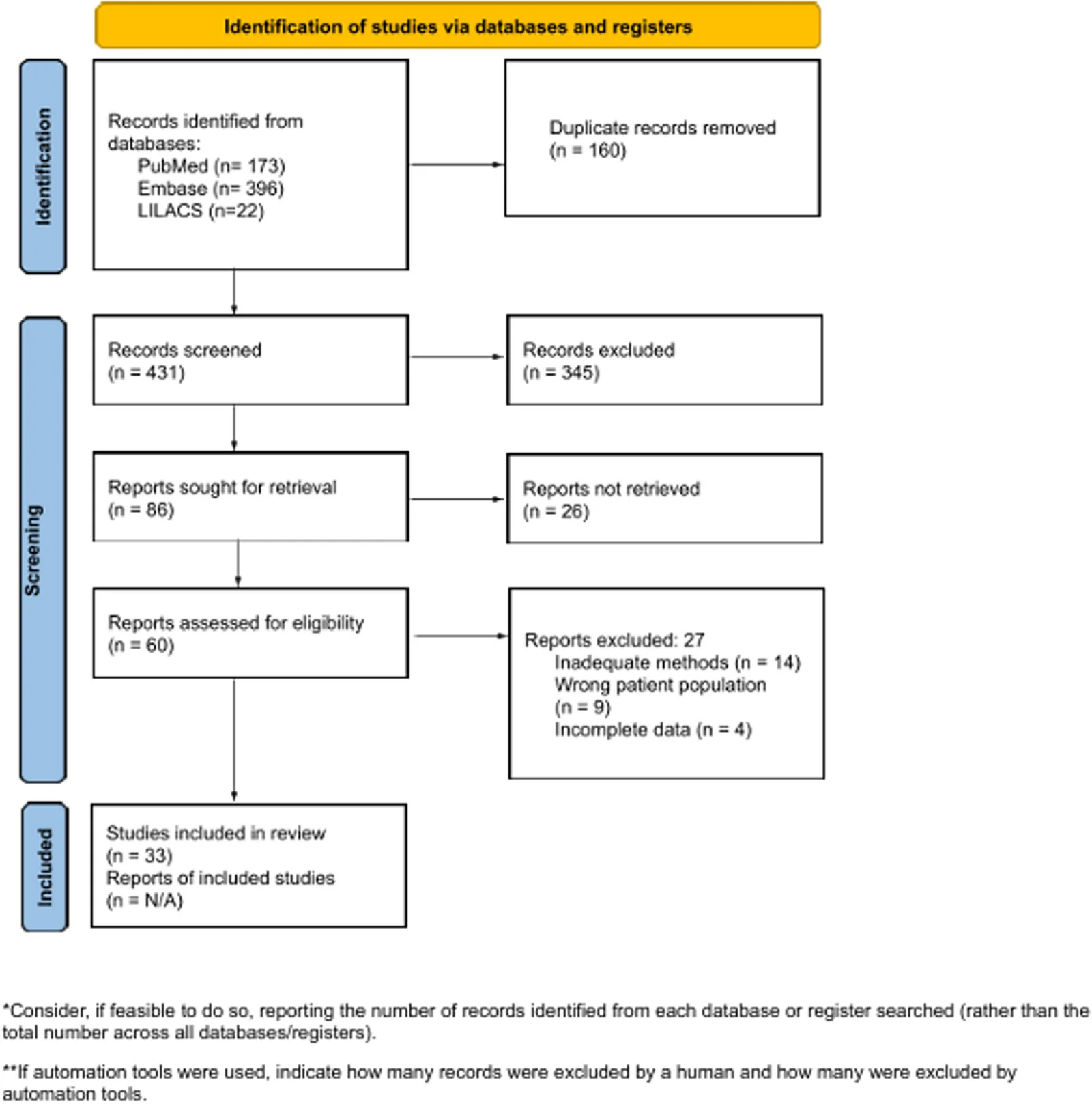
PRISMA flowchart of article exclusion during peer-review

As illustrated in Fig. 2, our systematic search identified 591 records from electronic databases (PubMed = 173, Embase = 396, and LILACS = 22). After removing 150 duplicates, 431 records remained for title and abstract screening; 345 were excluded for failing to meet the inclusion criteria. The remaining 86 reports were sought for retrieval, but 26 were not obtained. Subsequently, 60 full-text articles were assessed for eligibility, and 27 were excluded due to inadequate methods (*n* = 14), wrong patient population (*n* = 9), or incomplete data (*n* = 4). Ultimately, 33 studies met the inclusion criteria and were included in the review.

The included studies included a total of 17,100 participants, of whom 6,644 (38.8%) were positive for CD. Table 1 summarizes the key characteristics of the included studies, including study design, setting, population demographics, and reported electrocardiographic outcomes such as AF and other conduction abnormalities.

**Table 1:** Baseline Characteristics Of Included Studies

Author, year	Country	Type of Study	Sample size	Population	Number of Patients CD/Non-CD	Male Sex: n° (%) CD/Non-CD	Age, years: CD/Non-CD	Diabetes: n° (%) CD/Non-CD	Hypertension: n° CD/Non-CD	History Atrial Fibrillation: n° CD/Non-CD	Diagnosis of CD	Cardiac Form Only	Only HF Patients	Control Group
Abuhab et al., 2013	Brazil	Prospective cohort	577	ADHF admissions	CD: 58	33 (56.9) / 317 (61.1)	61.75 ± 4.2 / 68.25 ± 3.17	13 (22.4) / 236 (45.5)	N/A	18 (31.0) / 151 (29.1)	Serology, NS	Yes	Yes	CMP: Ischemic,
					Non-CD: 519									Idiopathic,
														Hypertensive, others
Acquatella et al., 1987	Venezuela	Cross-sectional	1,698	Community survey	CD: 775	N/A	47.3 ± 14.5 / 40.5 ± 15.5	N/A	N/A	N/A	CFT, IF or HA	No	No	No CMP
					Non-CD 923									
Arce et al., 2012	Argentina	Retrospective cohort	360	Pacemaker patients	CD: 180	130 (72) / 108 (60)	66.29 ± 7.01 / 75.3 ± 7.11	16 (9) / 57 (32)	87 (48) / 130 (72)	18 (10) / 12 (6.6)	IF, HA or ELISA	Yes	No	CMP: Ischemic
					Non-CD: 180									
Ballinas et al., 2003	México	Cross-sectional	58	Only CMP	CD: 26	N/A	47.8 ± 13.5 / 48.5 ± 12.9	N/A	N/A	N/A	Serology, NS	Yes	NA	CMP: Dilated
					Non-CD: 32									
Bestetti et al., 1997	Brazil	Prospective cohort	125	Outpatient CMP clinic	CD: 75	42 (56) / 35 (70)	55 ± 14 / 59 ± 14	N/A	N/A	N/A	CFT	Yes	Yes	CMP: Ischemic,
					Non-CD: 50									Idiopathic,
														Hypertensive, others
Borges-Pereira et al., 2001	Brazil	Cross-sectional	14,709	Community survey	CD: 270	97 (35.9) / 6.296 (43.6)	46.47 ± 18.18 / 28.39 ± 19.79	N/A	32 (16.7) / 28 (14.6)	N/A	IF, HA and ELISA	No	No	No CMP
					Non-CD: 14.439									
Bravo Tobar et al., 2011	Venezuela	Cross-sectional	1,372	Outpatient clinic and	CD: 200	75 (37.5) / 463 (39.5)	37.39 ± 42.16 / 22.84 ± 26.90	N/A	N/A	24 (36.9) / 33 (34)	ELISA, IF and DA	No	No	No CMP
				ER	Non-CD: 1172									
Carod-Artal et al., 2005	Brazil	Prospective cohort	244	Stroke patients	CD: 94	39 (41.4) / 88 (58.6)	56.31 ± 14.18 / 61.59 ± 8.88	16 (17) / 40 (26.66)	58 (61.70) / 132 (88)	13 (13.83) / 8 (5.33)	IF and HA	No	No	No CMP
					Non-CD: 150									
Carod-Artal et al., 2007	Brazil	Prospective cohort	220	Stroke patients	CD: 80	40 (50) / 77 (55)	59.5 ± 11.4 / 60.1 ± 13.8	4 (5) / 37 (26.4)	49 (62) / 104 (74.3)	8 (10.1) / 11 (7.9)	IF and HA	No	No	No CMP
					Non-CD: 140									
Carod-Artal et al., 2011	Brazil	Prospective cohort	146	Stroke patients	CD: 86	31 (36) / 21 (35)	57.4 ± 14.6 / 61.1 ± 9.1	14 (16.2) / 14 (23.3)	54 (62.7) / 52 (86.7)	18 (33.1) / 3 (5)	IF and HA	No	No	No CMP
					Non-CD: 60									
Cerqueira-Silva et al., 2022	Brazil	Prospective cohort	535	Outpatient clinic	CD: 271	107 (39.4) / 139 (52.6)	55.5 ± 11.0 / 53.1 ± 14.5	47/264 (17.8) / 61/256 (23.8)	159/262 (60.7) / 181/256 (70.7)	16/205 (7.8) / 8/169 (3.6)	ELISA	Yes	Yes	No CMP
					Non-CD: 264									
Cougo Pinto et al., 2013	Brazil	Retrospective cohort	174	Stroke patients	CD: 24	14 (58.3) / 85 (56.7)	65.6 ± 13.1 / 63.6 ± 13.0	5 (20.8) / 44 (29.3)	18 (75.0) / 121 (80.7)	6 (25.0) / 28 (18.7)	IF and HA	No	No	No CMP
					Non-CD: 150									
Coura et al., 2013	Brazil	Case-control	106	Community survey	CD: 106	N/A	N/A	N/A	N/A	N/A	IF and ELISA	No	No	No CMP
					Non-CD: 106									
Da Matta et al., 2012	Brazil	Cross-sectional	790	Outpatient CMP clinic	CD: 329	176 (53.5) / 261 (56.6)	58.8 ± 11.4 / 58.0 ± 14.3	31 (9.5) / 62 (13.4)	139 (42.1) / 291 (63.1)	33 (10.0) / 32 (7.0)	IF, HA or ELISA	Yes	No	CMP: Dilated,
					Non-CD: 461									Hypertrophic and
														Restrictive
Dias, 1993	Brazil	Cross-sectional	301	Community survey	CD: 35	35 (100.0) / 266 (100.0)	N/A	N/A	N/A	N/A	IF and HA	No	No	No CMP
					Non-CD: 266									
Emilio De All et al., 2024	Argentina	Cross-sectional	79	Community survey	CD: 41	19 (46.0) / 13 (34.0)	42 ± 13 / 38 ± 14	5 (12) / 3 (8)	18 (44) / 10 (26)	N/A	IF, ELISA and HA	No	No	No CMP
					Non-CD: 38									
Goldbaum et al., 2004	Brazil	Cross-sectional	949	Survey, industry	CD: 373	N/A	35.5 ± 34.0 / 34.2 ± 33.0	N/A	N/A	N/A	IF, HA and CFT	No	No	No CMP
				Workers	Non-CD: 576									
Issa et al., 2010	Brazil	Prospective cohort	456	Outpatient CMP clinic	CD: 68	41 (60.3)/ 280 (72.2)	50.4 ± 1.2 / 50.2 ± 0.6	N/A	N/A	8 (11.5) / 70 (18)	IF, HA or ELISA	Yes	Yes	CMP: Idiopathic, hypertensive, others
					Non-CD: 388									
Jorge et al., 2003	Brazil	Cross-sectional	3,056	ECG abnormality	CD: 1013	N/A	N/A	N/A	N/A	87 (8.59) / 63 (3.08)	Serology, NS	Yes	No	No CMP
					Non-CD: 2043									
Maguire et al., 1983	Brazil	Cross-sectional	644	Community survey	CD: 346	157 (45,37) / 132 (45,6)	34.3 ± 16.9 / 31.6 ± 18.7	N/A	N/A	N/A	CFT	No	No	No CMP
					Non-CD: 298									
Martineli Filho et al., 2018	Brazil	Retrospective cohort	426	Outpatient CMP clinic	CD: 115	75 (65.2) / 201 (64.6)	56.7 ± 11.8 / 63.2 ± 11.5	13 (11.3) / 115 (36.9)	38 (33.0) / 194 (62.37)	29 (25.2)/ 48 (15.43)	Serology, NS	No	No	CMP: Dilated and Ischemic
					Non-CD: 311									
Oliveira et al., 2021	Colombia	Cross-sectional	80	Outpatient clinic	CD: 40	25 (62.5) / 25 (62.5)	62.4 ± 2.6 / 63.3 ± 2.4	18 (45.0) / 24 (60.0)	27 (67.5) / 33 (82.5)	10 (25.0)/ 12 (30.0)	ELISA and IF	Yes	Yes	No CMP
					Non-CD: 40									
Parada et al., 1989	Venezuela	Cohort	200	Only CMP	CD: 96	N/A	N/A	N/A	N/A	N/A	Serology, NS	Yes	No	CMP: Dilated
					Non-CD: 104									
Pereira et al., 2002	Brazil	Cross-sectional	330	Community survey	CD: 189	84 (44.4) / 58 (41.1)	54 ± 15.6 / 52 ± 14.8	N/A	26 (13.8) / 19 (13.5)	1 (0.5) / 0 (0)	IF, HA and ELISA	No	No	No CMP
					Non-CD: 141									
Pereira Nunes et al., 2010	Brazil	Prospective cohort	287	Only dilated CMP	CD: 224	137 (61) / 38 (60)	48.3 ± 12 / 49.6 ± 15.9	N/A	N/A	11 (5) / 2 (3)	Serology, NS	Yes	Yes	CMP: Idiopathic,
					Non-CD: 63									Dilated
Pompilio et al., 2005	Brazil	Cross-sectional	240	Outpatient ID clinic	CD: 120	N/A	N/A	N/A	43 (35.8) / 20 (16.7)	0 (0) / 3 (2.5)	IF, HA or ELISA	No	No	No CMP
				and healthy controls	Non-CD: 120									
Ribeiro et al., 2013	Brazil	Retrospective cohort	987	Blood bank screening	CD: 499	261 (52) / 241 (49)	48.25 ± 2.8 / 49.5 ± 2.67	27 (5) / 24 (5)	113 (23) / 119 (24)	N/A	IF, HA or ELISA	No	No	No CMP
					Non-CD: 488									
Ribeiro et al., 2014	Brazil	Cohort	1,462	Community survey,	CD: 557	181 (32.5) / 391 (43.2)	68.5 ± 1.67 / 67.5 ± 1.67	57 (10.3) / 152 (16.8)	N/A	N/A	HA and ELISA	No	No	No CMP
				≥ 60 years old	Non-CD: 905									
Romano et al., 2020	Brazil	Cross-sectional	40	Outpatient clinic	CD: 20	6 (30) / 11 (55)	53 ± 12 / 48 ± 10	1 (5) / 0 (0)	N/A	2 (10) / 0	Serology, NS	Yes	No	No CMP
					Non-CD: 20									
Sierra-Johnson et al., 2005	Mexico	Case-control	128	Hospital-based CMP	CD: 51	25 (49) / 50 (65)	55 ± 10 / 42 ± 17	8 (16) / 10 (13)	11 (21) / 9 (12)	N/A	IF and ELISA	Yes	Yes	CMP: Dilated
				Clinic	Non-CD: 77									
Vieira de Melo et al., 2019	Brazil	Retrospective cohort	108	Outpatient CMP clinic	CD: 52	32 (61.5) / 38 (67.9)	59.2 ± 10.9 / 56.8 ± 13.4	11 (21.2) / 13 (23.2)	N/A	6 (11.5) / 4 (7.1)	Serology, NS	Yes	Yes	CMP: Ischemic,
					Non-CD: 56									Idiopathic,
														Hypertensive, others
Vilas-Boas et al., 2012	Brazil	Prospective cohort	301	Outpatient CMP clinic	CD: 222	143 (64) / 72 (91)	54 ± 14 / 64 ± 11	N/A	N/A	N/A	Serology, NS	Yes	Yes	CMP: Ischemic
					Non-CD: 79									
Yacoub et al., 2003	Honduras	Case-control	133	Cardiology clinic and	CD: 88	51 (57.9) / 25 (55.5)	44.8 ± 12.7	N/A	N/A	N/A	IF and ELISA	No	No	No CMP
				blood donors	Non-CD: 45									

Note: *CD*: Chagas Disease; *Non-CD*: No Chagas Disease; *NS*: Not specified; *CFT*: Complement Fixation Technique; *IF*: Immunofluorescence; *HA*: Hemagglutination; *ELISA*: Enzyme Linked Immuno Sorbent Assay; *HF*: Heart Failure; *CMP*: Cardiomyopathy; *ADHF*: Acute Decompensated HF Admissions; ER; ECG; *Mean ± standard deviation

### Data extraction

This meta-analysis was conducted and reported in accordance with the PRISMA 2020 (Preferred Reporting Items for Systematic Reviews and Meta-Analyses) [8]. Data extraction was performed independently by three investigators (F.L., G.R., and A.C.) using a standardized, prespecified data collection form. Extracted variables included study characteristics (first author, publication year, study design, diagnostic criteria for ChMM, sample size, geographic location, and study period), population characteristics (presence and type of cardiomyopathy, heart failure status, and composition of the control group), and electrophysiological outcomes of interest. These outcomes comprised AF and conduction system abnormalities, including right bundle branch block (RBBB), left anterior fascicular block, and AVB (grades I–III). Discrepancies between reviewers were resolved by consensus and, when necessary, adjudicated by a fourth investigator. The study protocol was prospectively registered in the International Prospective Register of Systematic Reviews (PROSPERO; CRD420251005184).

### Statistical analysis

For each outcome, odds ratios (ORs) with corresponding 95% confidence intervals (CIs) were calculated as the summary measures of effect. Pooled estimates were derived using a random-effects model based on the Mantel–Haenszel method to account for anticipated clinical and methodological heterogeneity across studies. Statistical heterogeneity was assessed using Cochran’s Q test and quantified with the I² statistic, with thresholds of approximately 25%, 50%, and 75% indicating low, moderate, and high heterogeneity, respectively. Prespecified subgroup analyses were conducted according to cardiomyopathy subtype when sufficient data were available. Publication bias was evaluated visually using funnel plots and formally assessed with Egger’s regression test when at least 10 studies contributed to an outcome. All statistical tests were two-sided, and p-values < 0.05 were considered statistically significant. Analyses were performed using RStudio (version 4.5.1) with dedicated meta-analysis packages.

### Risk of bias assessment

Risk of bias was independently assessed by two reviewers (AA and FL) using the Newcastle Ottawa Scale (NOS) [9] for non-randomized studies, covering three domains: selection, comparability, and outcome assessment. Disagreements were resolved through discussion. The results were summarized as low, moderate, or high risk of bias. Publication bias was assessed through funnel plots and Egger’s regression test.

## Results

### AF in patients with ChCM

**Fig. 3:**
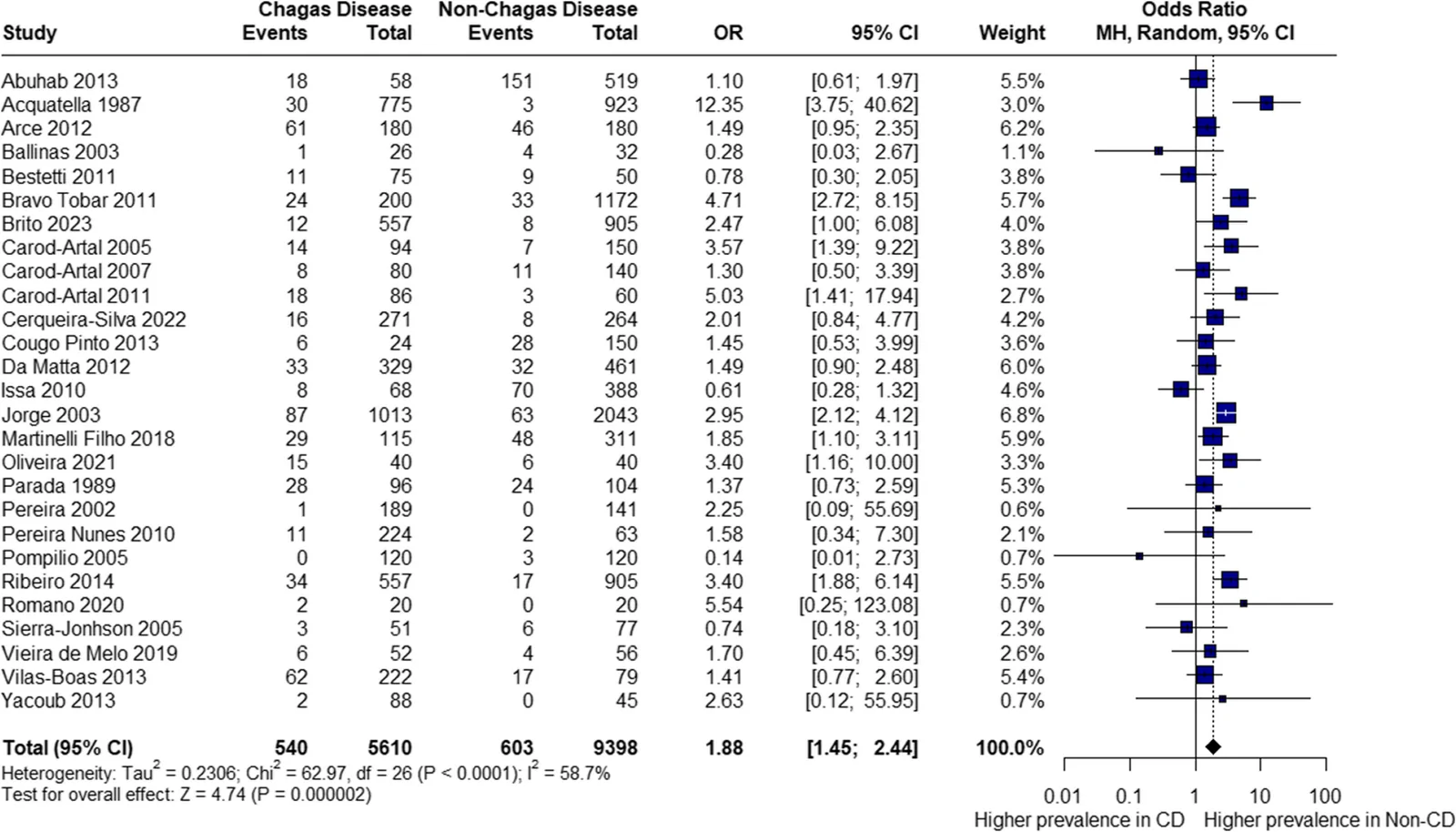
Prevalence of atrial fibrillation in Chagas disease versus controls

The prevalence of AF was reported in 27 of the 33 included studies, with a total of 15,088 patients. The prevalence of AF was remarkably higher among individuals with CD (9.92%) compared to those without CD (6.61%), corresponding to a pooled OR of 1.88 (95% CI: 1.45–2.44; *p* = 0.000002; I² = 58.7%) (Fig. 3). The non-CD comparator group comprised a heterogeneous population, including both healthy individuals and patients with structural heart disease. This heterogeneity likely reflects real-world epidemiology but may have contributed to between-study variability and limits causal inference regarding disease-specific mechanisms. The primary analysis evaluated the prevalence of AF and conduction abnormalities in individuals with CD compared with non-Chagas controls. In contrast, prespecified subgroup analyses examined patients with established ChCM relative to other cardiomyopathy subtypes.

### AF in patients with heart disease

**Fig. 4:**
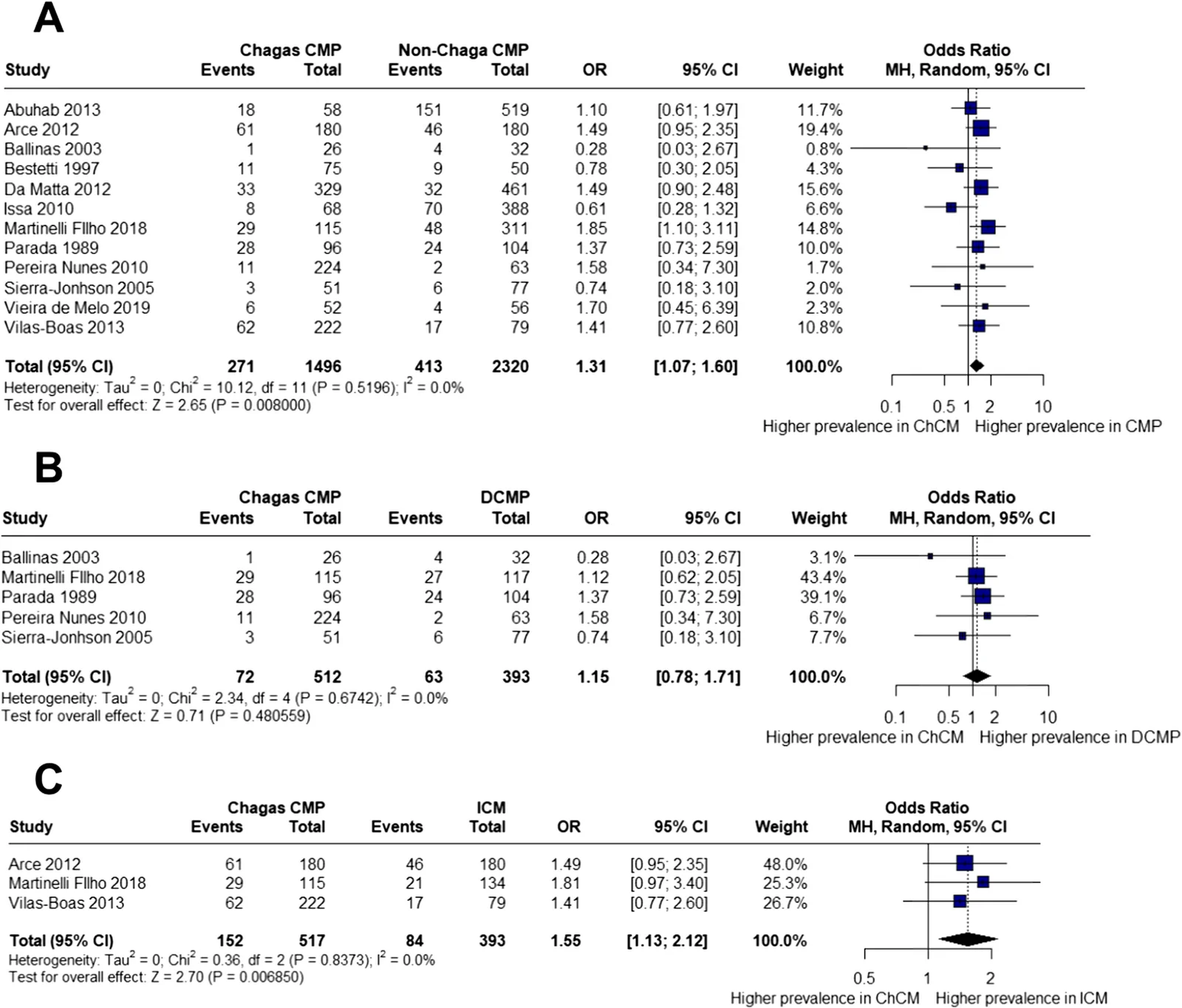
Prevalence of atrial fibrillation in Chagas cardiomyopathy versus other cardiomyopathy subtypes: **A** non-Chagas cardiomyopathy; **B** dilated cardiomyopathy; **C** ischemic cardiomyopathy

To control for potential confounding by underlying heart disease, we performed a subgroup analysis of 11 studies in which both the CD and non-CD groups consisted entirely of patients with cardiomyopathy, being the ChCM and non-CD cardiomyopathy groups. In this subset, AF remained significantly more prevalent among patients with ChCM compared to those with other forms of cardiomyopathy, with a pooled OR of 1.31 (95% CI: 1.07–1.60; *p* = 0.008; I² = 0.0%) (Fig. 4A).

### ICM vs. ChCM

Within the subgroup of patients with primary heart disease, we further stratified the analysis by etiology: ICM (*n* = 3 studies) and DCM (*n* = 5 studies). A significantly higher prevalence of AF was observed among patients with ChCM compared to those with ICM, with an OR of 1.55 (95% CI: 1.13–2.12; *p* = 0.00685; I² = 0.0%; Fig. 4C). In contrast, no significant difference was found when comparing ChCM to DCM with an OR of 1.15 (95% CI: 0.78–1.71; *p* = 0.480559; I² = 0.0%; Fig. 4B).

### Patients with heart failure (HF) vs. CD

**Fig. 5:**
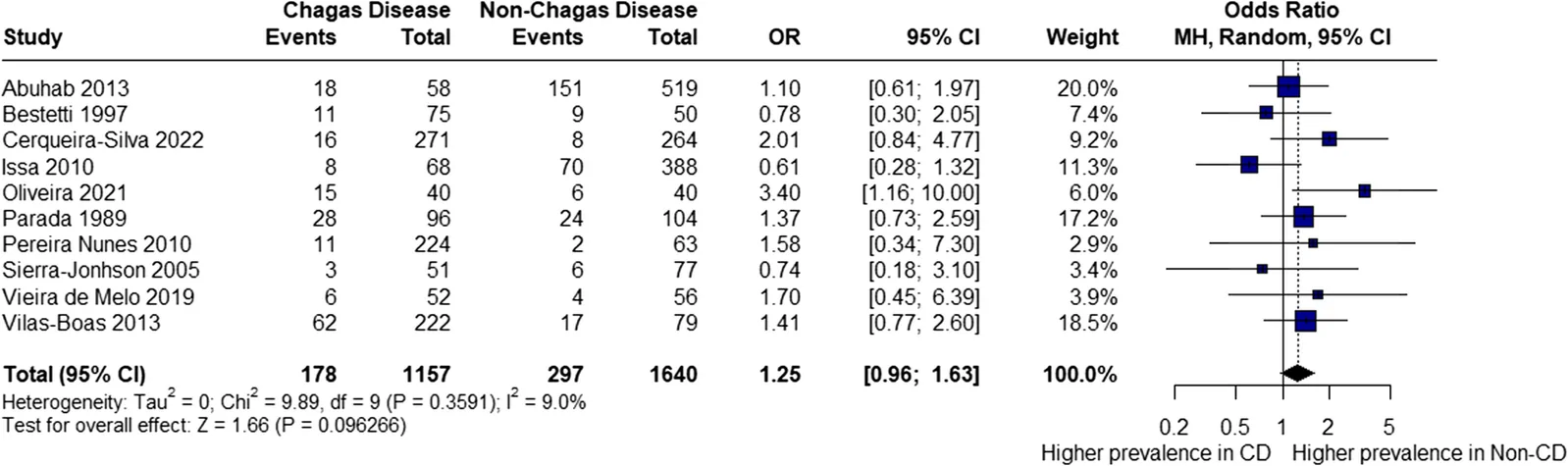
Prevalence of atrial fibrillation in Chagas disease patients with heart failure versus non-Chagas heart failure patients

In a subgroup analysis limited to patients with HF, no statistically significant difference in AF prevalence was observed between those with CD and those without, with an OR of 1.25 (95% CI: 0.96–1.63; *p* = 0.096266; I² = 9.0%; Fig. 5).

### Second-degree AVB

**Fig. 6:**
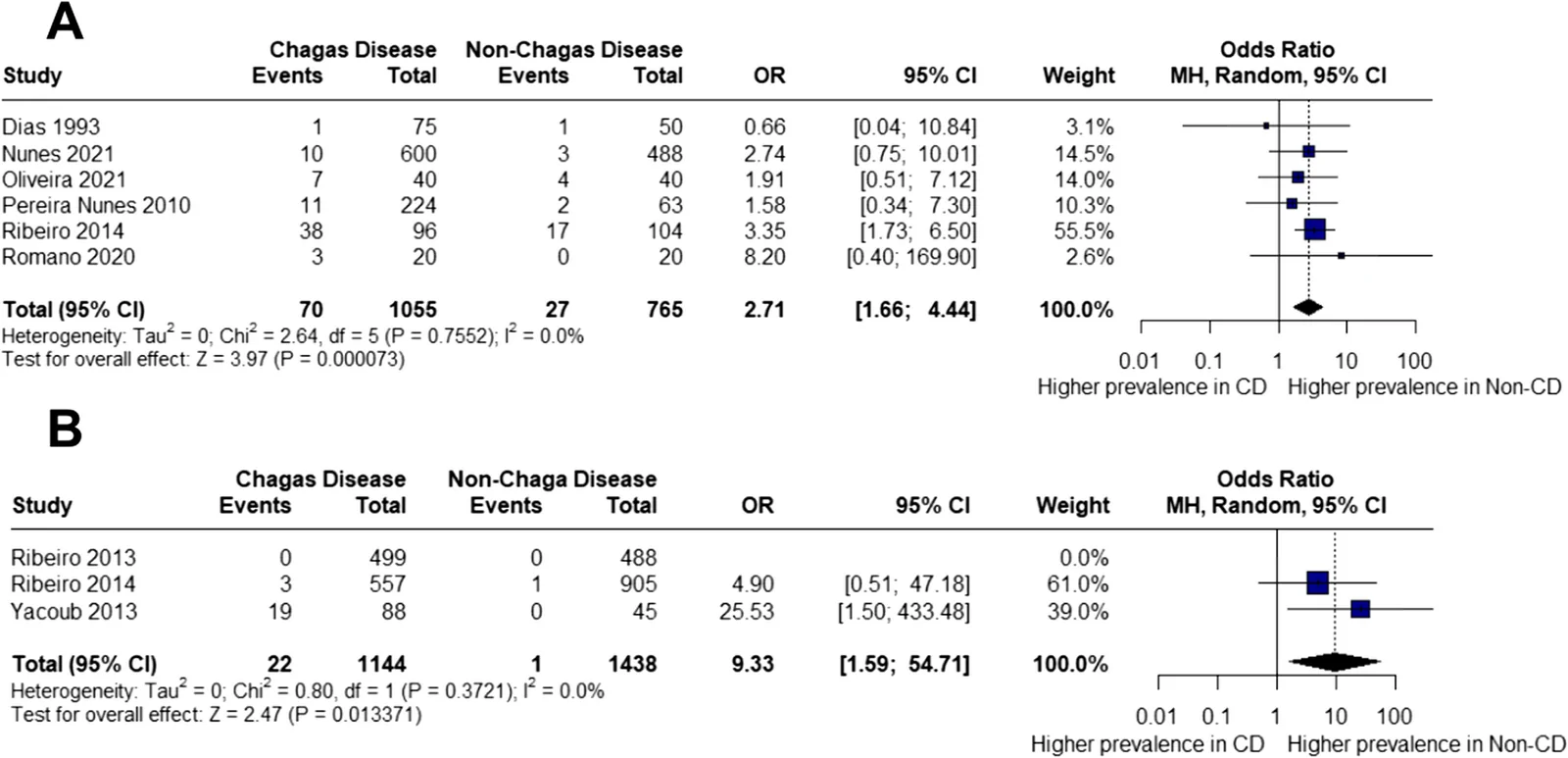
Prevalence of atrioventricular block in Chagas disease versus controls: **A** first-degree atrioventricular block; **B** third-degree atrioventricular block

The analysis of second-degree AVB included a total of 6 studies, which demonstrated a significantly higher prevalence in patients with CD compared to controls, with an OR of 2.55 (95% CI: 1.13–5.78; *p* = 0.024918; I² = 5.3%; Fig. 6A).

### Third-degree AVB

Although reported in fewer studies, third-degree AVB was significantly more frequent among patients with CD than in controls, with an OR of 2.10 (95% CI: 1.08–4.07; *p* = 0.027736; I² = 0.0%; Fig. 6B).

### RBBB

**Fig. 7:**
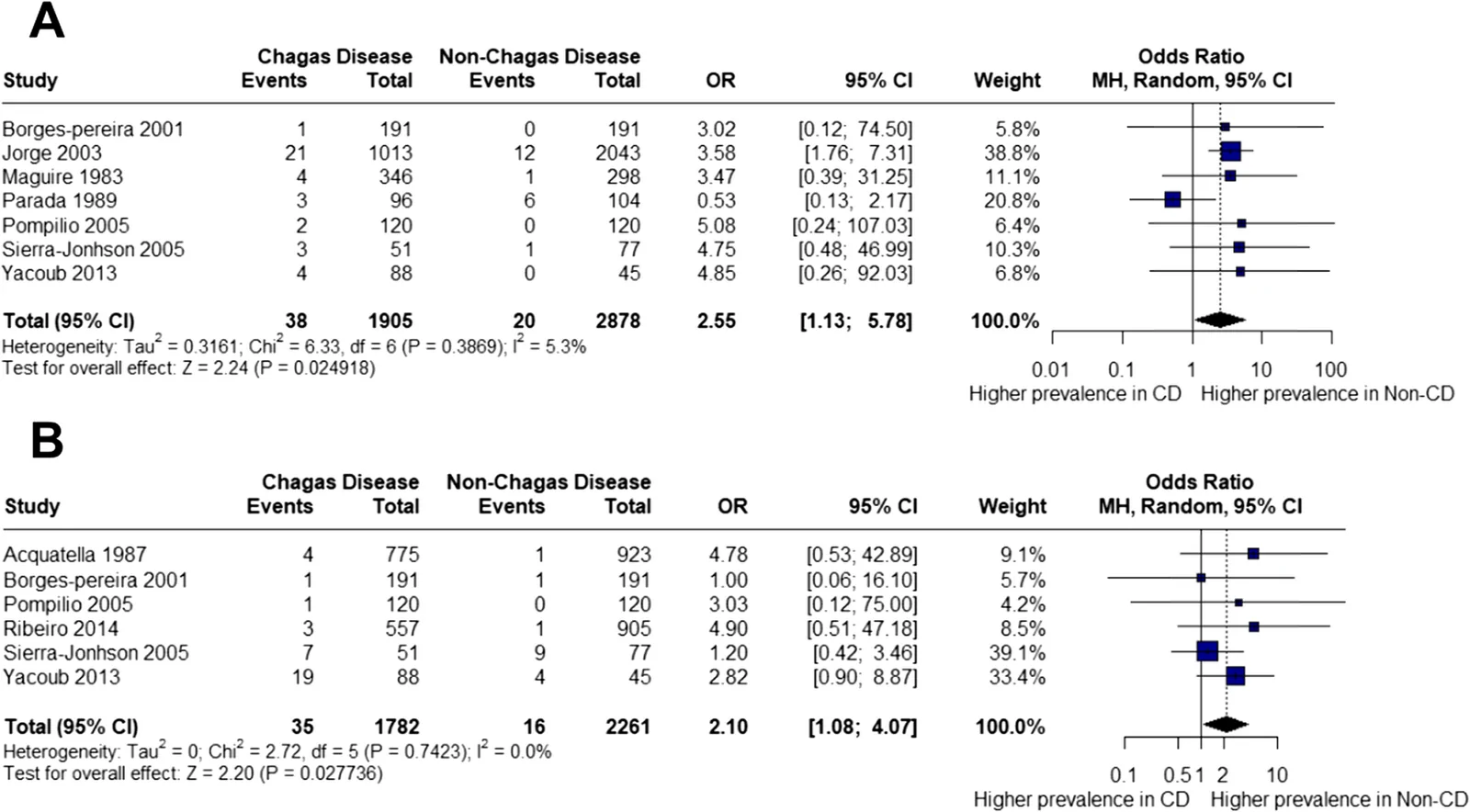
Prevalence of AVB in Chagas disease versus controls: **A** second-degree AVB; **B** third-degree AVB

The prevalence of RBBB was analyzed in studies comparing patients with CD and non-Chagas controls. The results demonstrated that RBBB was consistently more frequent among patients with CD, with an OR of 6.48 (95% CI: 4.41–9.52; *p* < 0.000001; I² = 73.4%; Fig. 7A).

### Isolated left anterior fascicular block (LAFB)

Isolated LAFB was also more frequent in patients with CD than in non-Chagas controls, with an OR of 3.06 (95% CI: 2.12–4.42; *p* < 0.000001; I² = 66.7%; Fig. 7B).

### RBBB combined with LAFB

When considering the combination of RBBB and LAFB, the prevalence was significantly higher in the CD group than in controls, with an OR of 9.79 (95% CI: 3.81–25.18; *p* = 0.000002; I² = 66.7%; Fig. 7C).

## Quality assessment

**Fig. 8:**
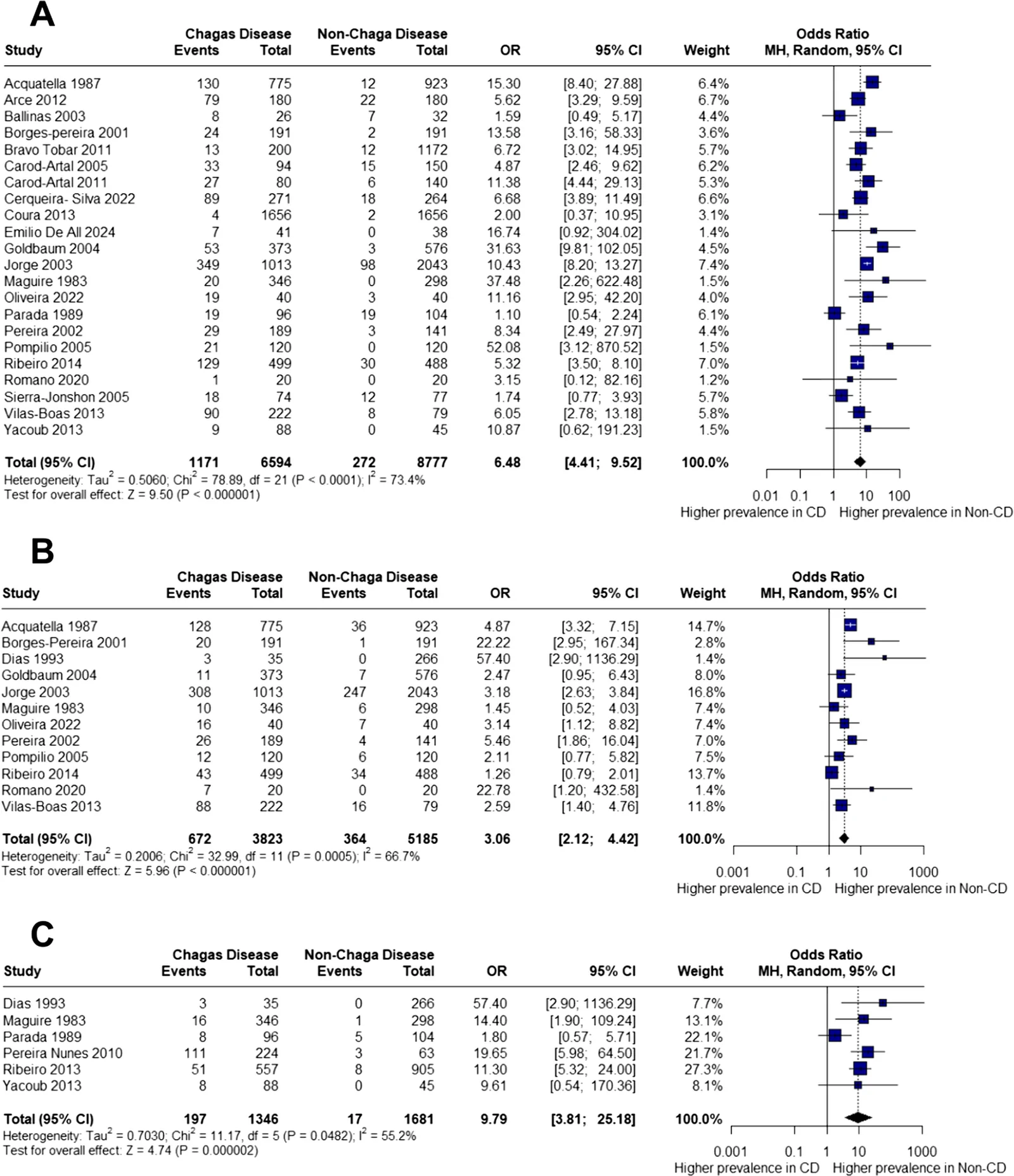
Prevalence of specific conduction abnormalities in Chagas disease versus controls: **A** isolated RBBB; **B** isolated LAFB; **C** combined RBBB and LAFB

**Fig. 9:**
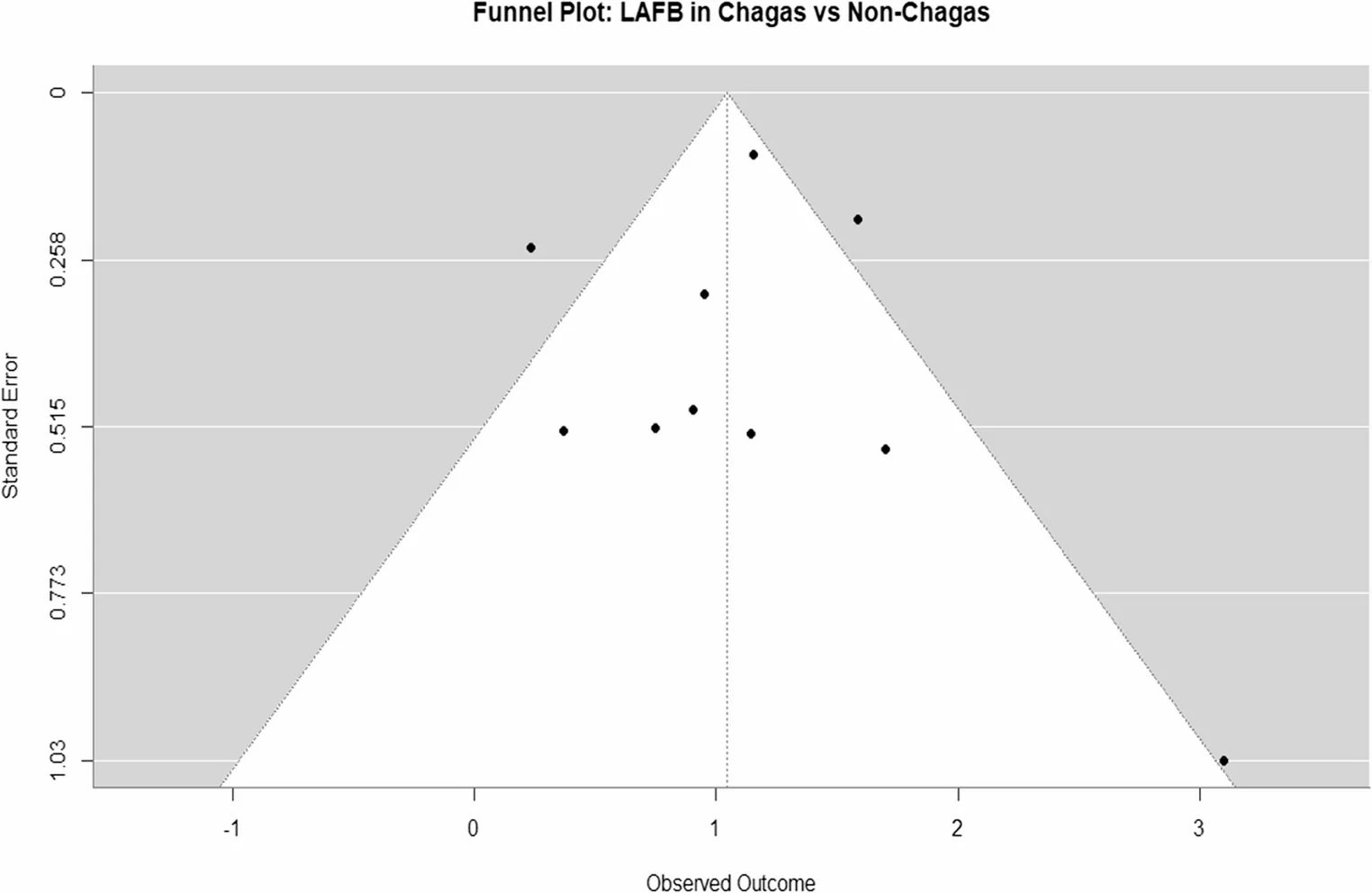
Funnel plot for the prevalence of right bundle branch block (RBBB) in Chagas disease versus controls. The visual symmetry suggests no significant publication bias, consistent with Egger’s test results

**Fig. 10:**
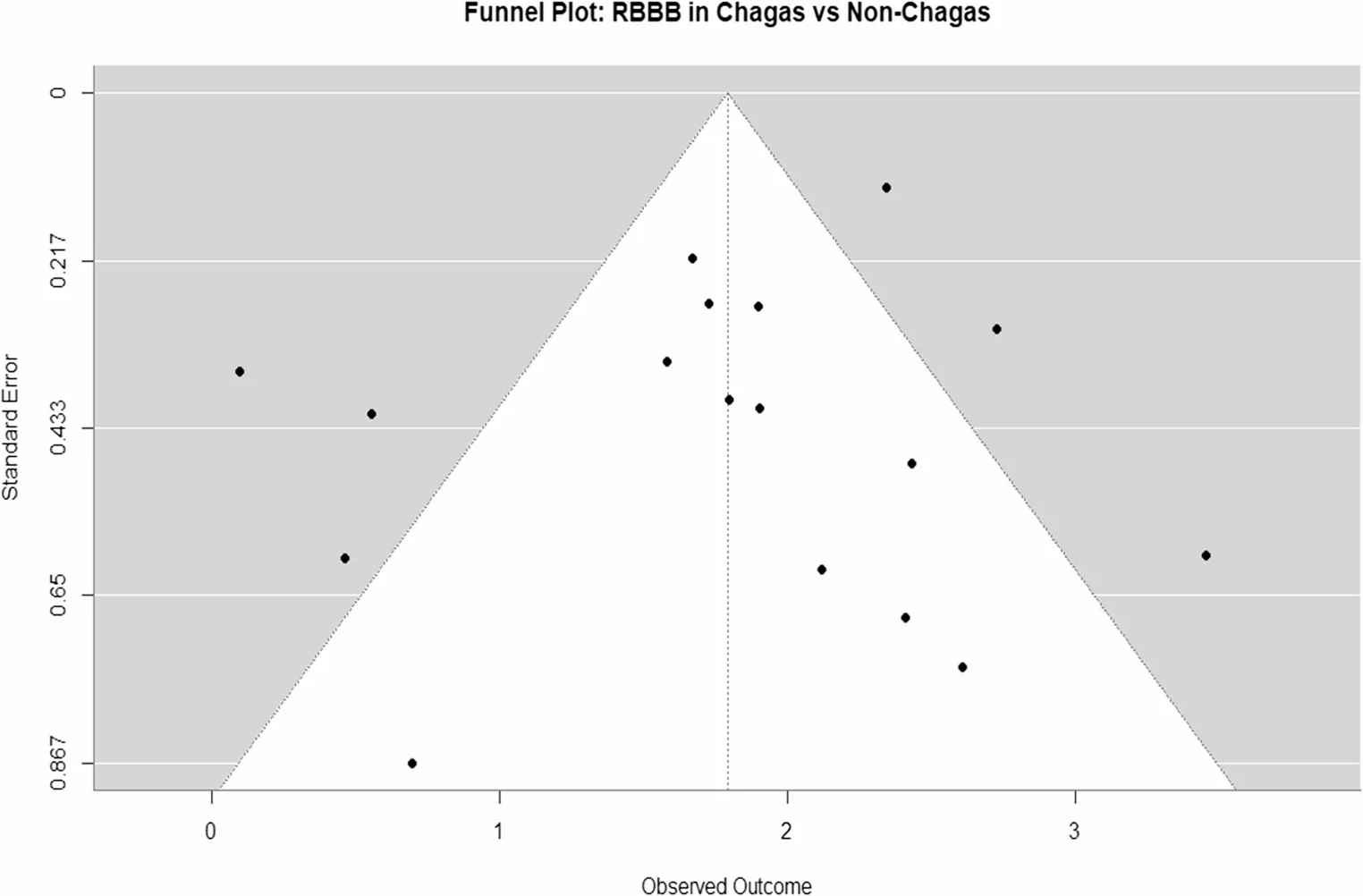
Funnel plot for the prevalence of combined RBBB with left anterior fascicular block in Chagas disease versus controls, showing no evidence of small-study effects or asymmetry

**Fig. 11:**
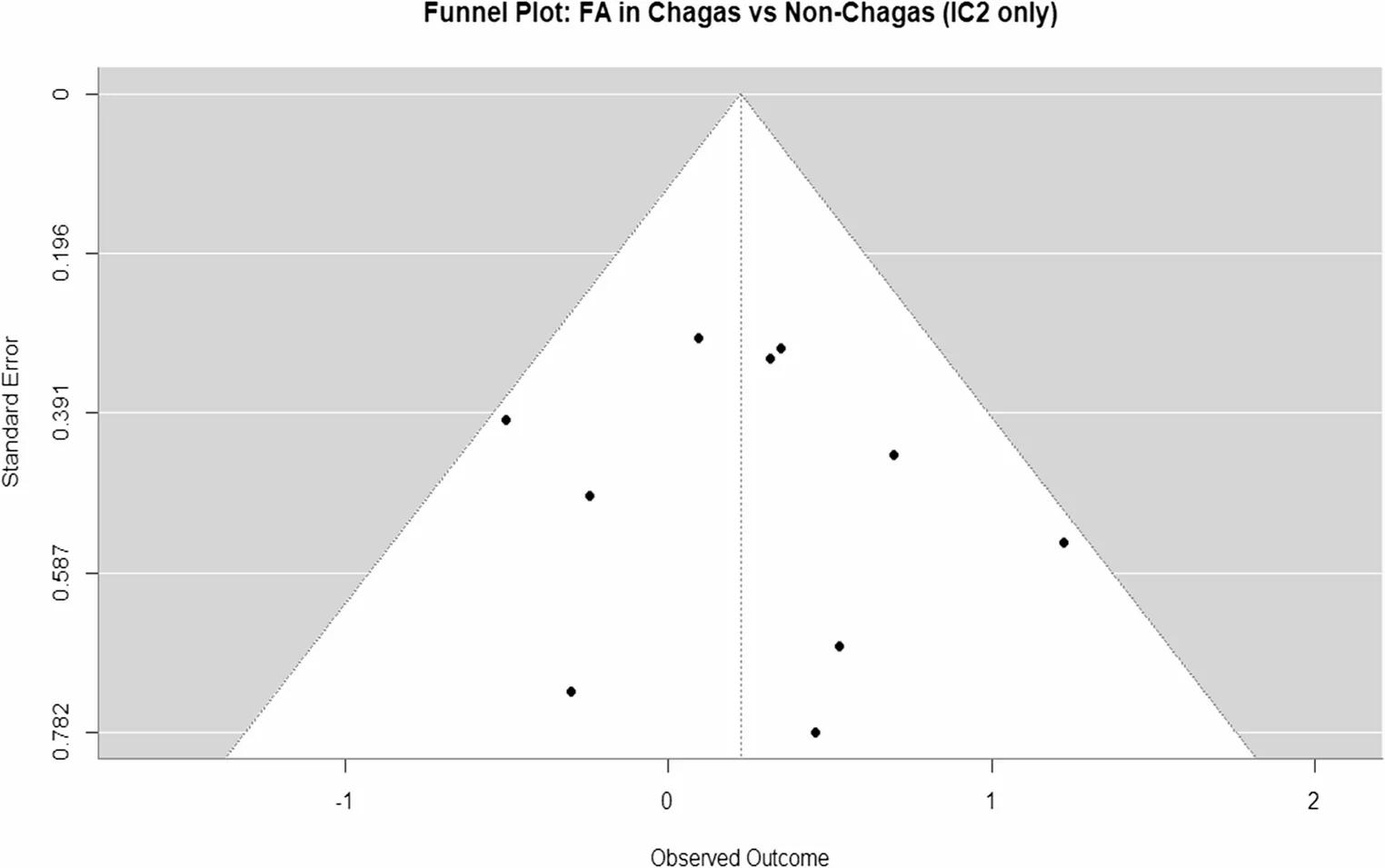
Funnel plot for first-degree atrioventricular block comparing Chagas and non-Chagas patients. The plot shows a balanced distribution of studies, indicating low publication bias

**Fig. 12:**
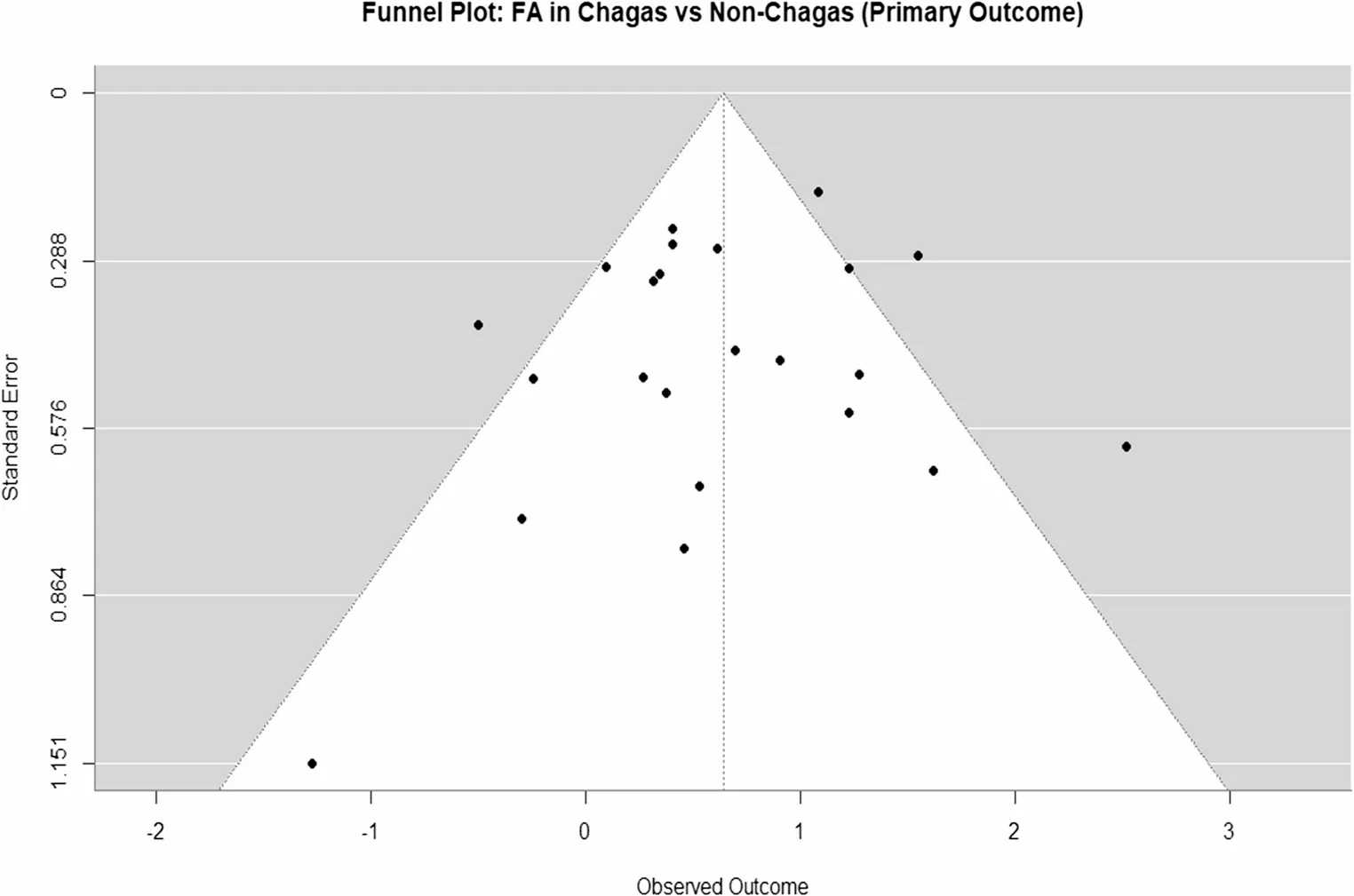
Funnel plot for atrial fibrillation prevalence in Chagas disease versus controls. The funnel appears symmetrical, supporting the robustness of the overall meta-analytic estimate

**Fig. 13:**
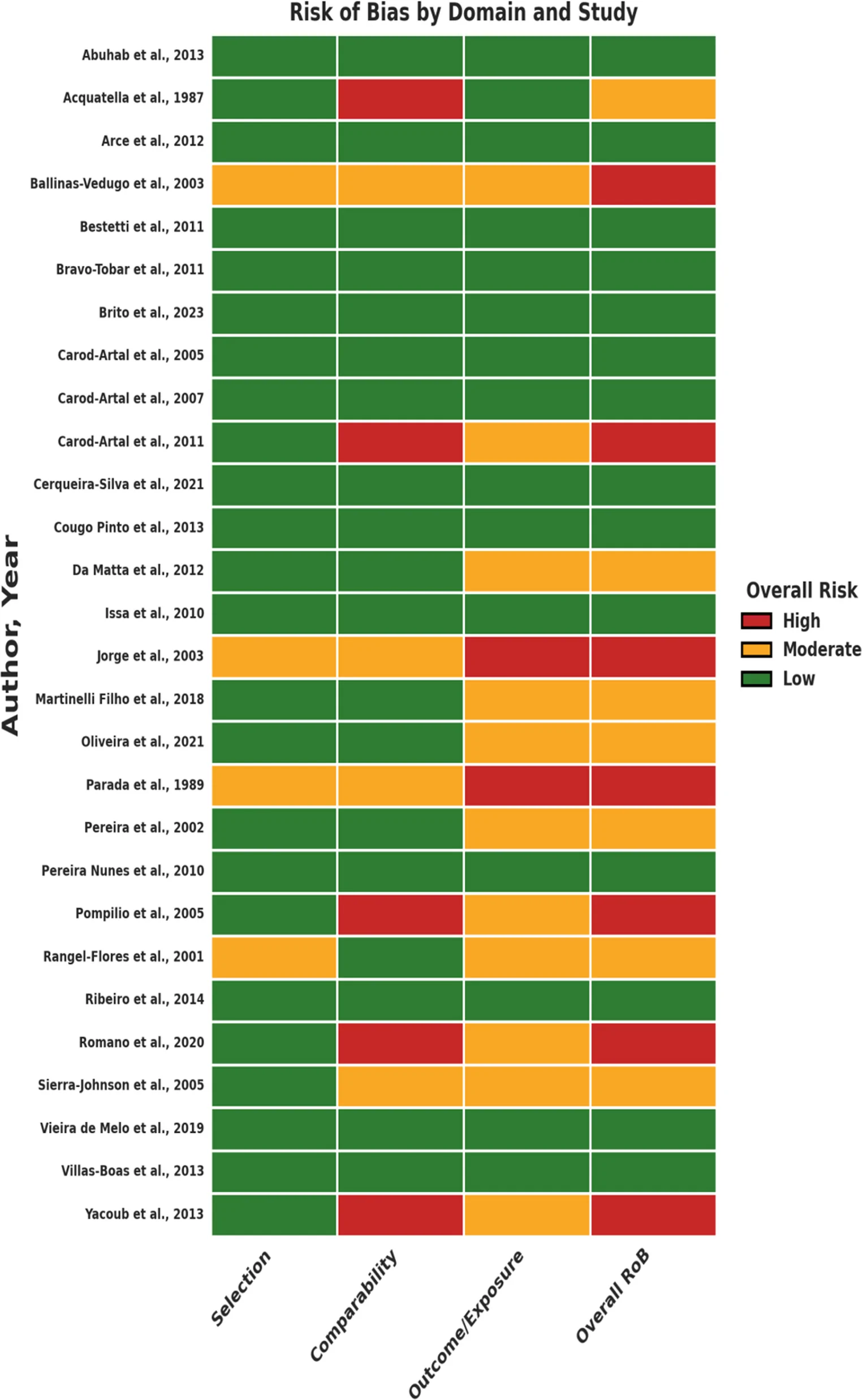
Risk of bias assessment of all included studies using the NOS

The risk of bias was assessed using the NOS, and detailed results are presented in (Fig. 13). Publication bias was assessed via funnel plot analysis and Egger’s linear regression test (Figs. 8, 9, 10, 11 and 12). The funnel plot for the primary outcome showed visual symmetry, and Egger’s test did not indicate significant publication bias (t = − 0.73; df = 26; *p* = 0.4715; bias estimate = − 0.44; SE = 0.61), suggesting no evidence of small-study effects or selective reporting. Overall, 9 studies were classified as having a low risk of bias, 11 as moderate risk, and 8 as high risk.

## Discussion

In this updated meta-analysis of 33 studies, we demonstrate that AF and conduction abnormalities are more prevalent among individuals with CD than in non-Chagas populations. Notably, most included studies compared seropositive individuals with heterogeneous control groups, frequently including healthy subjects and patients with varying degrees of structural heart disease. This population imbalance is a key source of heterogeneity. It underscores that our primary finding reflects the overall burden of arrhythmias associated with Chagas disease, rather than an isolated effect of Chagas cardiomyopathy.

### Comparison with previous meta-analyses

Our findings build upon and expand the conclusions of previous meta-analyses examining electrocardiographic abnormalities in CD. The systematic review by Rhanderson Cardoso et al. [6] was among the earliest quantitative syntheses on this topic, pooling data from 34 observational studies and demonstrating that CD was associated with markedly higher prevalence of RBBB, LAFB, and AVB than in seronegative controls. Cardoso and colleagues provided foundational epidemiologic evidence of conduction disturbances in CD, reporting pooled prevalences of approximately 26% for RBBB and 13% for LAFB, with combined RBBB + LAFB reaching nearly 10% [7]. However, their analysis did not differentiate between asymptomatic seropositive individuals and patients with overt ChCM, nor did it include comparisons with other cardiomyopathy etiologies.

Later, Rojas et al. [7] extended this work through a broader systematic review encompassing over 60,000 participants, confirming that Chagas patients exhibit increased odds of both conduction and repolarization abnormalities compared to the general population. Their results reinforced that involvement of the conduction system is a pathognomonic feature of ChCM. Nonetheless, their meta-analysis primarily compared CD with healthy or seronegative individuals and did not specifically address arrhythmia burden in patients with established cardiomyopathy or stratify outcomes by disease severity.

Our current meta-analysis updates and refines these earlier works in several important ways. First, by including 33 studies published through February 2025, we incorporate more recent data from both endemic and non-endemic settings, thereby capturing the evolving epidemiology of CD in migrant populations. Second, our analysis focuses on ChCM as a defined clinical phenotype, enabling direct comparison with other cardiomyopathies, including ischemic, dilated, and idiopathic forms, which prior meta-analyses did not examine. This approach highlights that the prevalence of AF, RBBB, and AVB in ChCM surpasses that observed in other structural heart diseases, suggesting a more diffuse and aggressive arrhythmogenic substrate.

Third, our work provides a quantitative synthesis of multiple conduction disturbances (isolated RBBB, combined RBBB + LAFB, isolated LAFB, and AVB of varying degrees), integrating data that were previously fragmented or inconsistently reported. This granularity allows for a more comprehensive understanding of how conduction system damage progresses across the clinical spectrum of CD.

Finally, by aligning our results with the 2023 ESC Guidelines for the Management of Cardiomyopathies [10], we contextualize the arrhythmic manifestations of CD within the modern framework of cardiomyopathy classification. Unlike prior meta-analyses that focused primarily on descriptive epidemiology, our study bridges these findings to clinical implications, demonstrating that the high burden of arrhythmias and conduction disorders in ChCM supports its classification as a distinct non-ICM with significant electrophysiological impact.

Together, these advances position our analysis as a timely update that not only confirms earlier observations from Cardoso and Rojas [6, 7] but also extends them through modern stratification, methodological rigor, and clinical relevance.

### Pathophysiological considerations

The electrophysiological phenotype of ChCM arises from multifactorial myocardial injury driven by Trypanosoma cruzi infection. Chronic inflammation and immune-mediated damage promote progressive fibrosis of the conduction system, particularly involving the RBB, left anterior fascicle, and atrioventricular node. Histopathological studies demonstrating lymphocytic infiltration, myocyte necrosis, and patchy fibrotic replacement within the His–Purkinje network provide a mechanistic basis for the high prevalence of bundle branch and fascicular blocks observed in our analysis [11, 12].

Autonomic dysfunction further contributes to arrhythmogenesis in ChCM. Preferential parasympathetic denervation with relative sympathetic predominance alters atrial refractoriness and conduction velocity, facilitating AF and reentrant arrhythmias [13–15]. In parallel, microvascular dysfunction and recurrent vasospasm may induce localized ischemic injury and conduction delay, even in the absence of epicardial coronary disease, distinguishing ChCM from ischemic cardiomyopathy [16, 17].

In advanced stages, ventricular remodeling with chamber dilation, wall thinning, and extension of fibrosis toward the apical region, including aneurysm formation, creates a heterogeneous substrate characterized by low-voltage areas and fractionated electrograms, favoring reentrant circuits [4, 18]. Collectively, inflammation, fibrosis, autonomic imbalance, and microvascular injury interact to produce a diffuse arrhythmogenic milieu, explaining the frequent coexistence of AF, atrioventricular block, and BBB in ChCM [4, 19, 20].

### Clinical implications

The clinical implications of our findings are considerable. The strong association between ChCM and both AF and conduction abnormalities underscores the need for disease-specific management strategies that are not yet clearly addressed in current international guidelines. Despite CD accounting for a significant proportion of non-ICM cases in Latin America, neither the ESC 2020 on AF nor the AHA/ACC/HRS 2019 update provides tailored recommendations for anticoagulation or rhythm control in this population [21, 22].

AF in CD carries a particularly high risk of thromboembolic events, often exceeding that of other cardiomyopathies, due to the coexistence of atrial dilation, mural thrombi, and ventricular dysfunction [11, 20]. Observational studies have shown that the annual stroke risk in patients with ChCM and AF may reach 4–6%, even among those with low CHA₂DS₂-VASc scores [19]. Although our analysis confirms a higher prevalence of AF in ChCM, it does not evaluate thromboembolic outcomes or anticoagulation strategies. Therefore, recommendations regarding anticoagulation in this population should rely on existing guidelines and individualized clinical judgment. Prospective studies are needed to clarify stroke risk stratification and the optimal anticoagulation approach in patients with ChCM [4].

The 2023 ESC Guidelines for the Management of Cardiomyopathies [23] recognize arrhythmias as central manifestations of cardiomyopathy and recommend routine arrhythmia screening with resting ECG and 24-hour Holter monitoring. These guidelines also emphasize that anticoagulation in cardiomyopathy should follow CHA₂DS₂-VASc criteria but may require individualized risk assessment, especially in diseases with diffuse fibrosis such as Chagas.

Regarding rhythm management, the success rate of catheter ablation for AF in CD is lower than in other forms of structural heart disease, likely due to extensive atrial fibrosis and conduction heterogeneity [18]. Amiodarone remains the most effective antiarrhythmic drug, but its long-term toxicity limits use, emphasizing the need for alternative strategies, including early ablation or hybrid procedures. Conduction disturbances are also clinically relevant. The high prevalence of RBBB and AVB in ChCM necessitates periodic ECG monitoring. Progressive AVB is frequent and may lead to syncope or sudden cardiac death. Consequently, the ESC 2023 guidelines recommend that patients with symptomatic bradycardia or high-degree AVB be evaluated for pacemaker implantation, and that implantable cardioverter-defibrillators (ICDs) be considered in those with reduced ejection fraction or sustained ventricular arrhythmias [23, 24].

Finally, the coexistence of AF, AVB, and ventricular arrhythmias illustrates the diffuse electrical remodeling that characterizes ChCM. Our results, consistent with contemporary guidelines, indicate that management should not rely solely on general cardiomyopathy algorithms but should incorporate disease-specific risk stratification, early anticoagulation, and proactive pacing strategies to reduce morbidity and mortality in this neglected condition.

### Strengths and limitations

This meta-analysis has several significant limitations. First, substantial heterogeneity in baseline characteristics existed between CD and non-Chagas-disease populations. Many studies compared seropositive individuals with younger and healthier seronegative controls, whereas others included patients with advanced cardiomyopathy, heart failure, or stroke. These differences, particularly in age, comorbidity burden, and disease severity, are well-established determinants of AF prevalence and could not be adequately adjusted for due to the absence of individual patient-level data.

Second, reporting of AF characteristics was inconsistent across studies. Most cohorts did not distinguish between paroxysmal, persistent, or permanent AF, and detection methods varied widely, ranging from a single resting electrocardiogram to prolonged ambulatory monitoring. Variability in surveillance intensity likely led to underdetection of AF in some populations and introduced outcome misclassification, contributing to between-study heterogeneity.

Third, information on antiarrhythmic drug use was sparsely reported and highly heterogeneous, precluding meaningful comparison across studies. As pharmacologic treatment may influence both AF prevalence and detection, this represents an additional source of unmeasured confounding.

Despite these limitations, this study has notable strengths. It represents the most comprehensive and up-to-date synthesis of evidence on AF and conduction abnormalities in ChCM, incorporating 33 studies published through February 2025. The inclusion of cohorts from both endemic and non-endemic regions enhances external validity and reflects the evolving global epidemiology of CD driven by migration.

Methodological rigor further strengthens the findings. A comprehensive, PRISMA-compliant search strategy was applied across multiple databases, with standardized data extraction and systematic risk-of-bias assessment using the NOS tool. Publication bias was formally evaluated using funnel plots and Egger’s test, with no evidence of significant small-study effects. Importantly, unlike prior reviews, this analysis focused specifically on ChCM as a distinct clinical phenotype and incorporated prespecified subgroup comparisons among ischemic, dilated, and idiopathic cardiomyopathies, thereby enabling a refined characterization of disease-specific electrophysiological patterns.

Overall, although residual confounding and heterogeneity cannot be entirely excluded, this meta-analysis provides a robust, clinically relevant synthesis that advances the epidemiologic and electrophysiologic understanding of ChCM and establishes a strong foundation for future prospective, phenotype-specific investigations.

## Conclusion

The results of this meta-analysis highlight the importance of ChCM’s distinctiveness within the spectrum of structural heart disease, particularly with respect to the genesis of arrhythmias. Investing in specific management strategies for this group, from early electrocardiographic screening to targeted treatment protocols, could represent a significant advance in reducing morbidity and mortality among the millions of patients affected by CD in the Americas. These findings emphasize the importance of systematic electrocardiographic screening and careful rhythm monitoring in patients with Chagas disease. Future prospective studies are needed to clarify thromboembolic risk stratification and to inform disease-specific management strategies.

## Supplementary information

Below is the link to the electronic supplementary material.

Supplementary Material 1

## Data Availability

The data used in this meta-analysis were extracted from publicly available sources, including published randomized controlled trials. All relevant data supporting the findings of this study are included within the manuscript and supplementary materials. Due to the nature of this analysis, no new primary data were generated. Additional details can be made available upon reasonable request to the corresponding author.

## Abbreviations

ACC: American College of Cardiology
AF: Atrial Fibrillation
AHA: American Heart Association
AV: Atrioventricular
AVB: Atrioventricular Block
BBB: Bundle Branch Blocks
CD: Chagas Disease
ChCM: Chagas cardiomyopathy
CI: Confidence Intervals
DCM: Dilated Cardiomyopathy
ECG: Electrocardiograph
ESC: European Society of Cardiology
HRS: Heart Rhythm Society
ICDs: Implantable Cardioverter defibrillators
ICM: Ischemic Cardiomyopathy
IDCM: Idiopathic Cardiomyopathy
LAFB: Left Anterior Fascicular Block
NOS: Newcastle Ottawa Scale
OR: Odds Ratios
RBBB: Right Bundle Branch Block
